# Active immunization combined with cisplatin confers enhanced therapeutic protection and prevents relapses of HPV-induced tumors at different anatomical sites

**DOI:** 10.7150/ijbs.56644

**Published:** 2022-01-01

**Authors:** Bruna Felício Milazzotto Maldonado Porchia, Luana Raposo de Melo Moraes Aps, Ana Carolina Ramos Moreno, Jamile Ramos da Silva, Mariângela de Oliveira Silva, Natiely Silva Sales, Rubens Prince dos Santos Alves, Clarissa Ribeiro Reily Rocha, Matheus Molina Silva, Karine Bitencourt Rodrigues, Tácita Borges Barros, Roberta Liberato Pagni, Patrícia da Cruz Souza, Mariana de Oliveira Diniz, Luís Carlos de Souza Ferreira

**Affiliations:** 1 Vaccine Development Laboratory, Department of Microbiology, Biomedical Sciences Institute, University of Sao Paulo, Sao Paulo, SP, Brazil; 2 ImunoTera Soluções Terapêuticas Ltda; 3 DNA Repair Laboratory, Department of Microbiology, Institute of Biomedical Sciences, University of Sao Paulo, Sao Paulo, Brazil; Porchia, BFMM and Aps, LRMM contributed equally to this work.

**Keywords:** Cancer, HPV, vaccine, gDE7, immunotherapy

## Abstract

The active immunotherapy concept relies on the use of vaccines that are capable of inducing antitumor immunity, reversion of the suppressive immunological environment, and long-term memory responses. Previously, antitumor vaccines based on a recombinant plasmid (pgDE7h) or a purified protein (gDE7) led to regression of early-established human papillomavirus (HPV)-associated tumors in a preclinical model. In this work, the anticancer vaccines were combined with cisplatin to treat HPV-induced tumors at advanced growth stages. The antitumor effects were evaluated in terms of tumor regression, induction of specific CD8^+^ T cells, and immune modulation of the tumor microenvironment. Acute toxicity induced by the treatment was measured by weight loss and histological alterations in the liver and kidneys. Our results revealed that the combination of cisplatin with either one of the tested immunotherapies (pgDE7h or gDE7) led to complete tumor regression in mice. Also, the combined treatment resulted in synergistic effects, particularly among mice immunized with gDE7, including activation of systemic and tumor-infiltrating E7-specific CD8^+^ T cells, tumor infiltration of macrophages and dendritic cells, and prevention of tumor relapses at different anatomical sites. Furthermore, the protocol allowed the reduction of cisplatin dosage and its intrinsic toxic effects, without reducing antitumor outcomes. These results expand our knowledge of active immunotherapy protocols and open perspectives for alternative treatments of HPV-associated tumors.

## Introduction

Human papillomavirus (HPV) infections cause almost 570,000 cases per year of invasive cervical cancer worldwide 1. Approximately 84% of cervical cancers and 88% of deaths caused by cervical cancer occurs in lower-resourced countries, of which 1.3% die from the disease before age 75, in the absence of competing causes of death 2. Cervical cancer represents the fourth most frequent cancer type and the third leading cause of cancer death in females 3, and continues to be a major public health problem affecting middle-aged women, particularly in less-resourced countries. Also, an increase in oropharyngeal cancer incidence rates (soft palate and uvula, tonsils, posterior pharyngeal wall, and tongue base) has been observed among the young adult population, mainly males, and mostly associated with HPV-16 4,5.

The possibility of preventing cancer development by controlling viral infection, led to the implementation of a worldwide vaccination program for certain types of HPV, including the most prevalent oncogenic genotypes. However, these prophylactic vaccines do not benefit patients with established tumors. Also, available cancer treatments are based on invasive methods with many adverse effects and are less effective for advanced-stage cancer. Moreover, surgery and chemotherapy are the first-choice treatments for advanced or invasive cervical cancer, despite a 5-year survival rate of less than 20% in treated patients 6. Furthermore, high doses of cisplatin, the most used chemotherapeutic agent, may negatively impact the antitumor responses in addition to inducing severe adverse side-effects 7. In this context, new and alternative therapies are needed to improve disease control and survival rates in those patients with tumors at more advanced stages.

Different treatments are available for patients with cervical cancer. Cisplatin is often administered as a treatment, with or without radiation therapy, for stage IB2 to IVA cervical cancer. However, its use is often associated with serious side-effects such as nephrotoxicity, despite the survival rates as an adjuvant platinum-based chemotherapy 8. Therefore, there is currently a growing interest in combined approaches based on chemo/radiotherapy and immunotherapy to treat solid tumors, and cancer therapeutic vaccines could play an important role in these settings. Over the last decade, important advances in tumor treatment were achieved by using passive immunotherapies. Monoclonal antibodies (mAbs) targeting immune checkpoints led to an intense wave of preclinical and clinical investigations that resulted in the clinical use of immunotherapy for different cancer types 9. Taking cervical cancer into account, mAbs targeting the extracellular domains of tyrosine kinase receptors (epidermal growth factor receptor (EGFR) and insulin-like growth factor receptor (IGFR) were approved for recurrent or metastatic cervical carcinoma 10. More recently, pembrolizumab was approved by the United States Food and Drug Administration (US FDA) for patients with recurrent or metastatic cervical cancer, or after chemotherapy for tumors expressing PD-L1. Despite the increase in overall survival rate, some studies reported relevant toxicity events related to the administration of these mAbs and variable clinical success rates 11.

Considering the induction of immune responses, antitumor vaccines have been used to stimulate T cells to recognize and destroy tumor cells, leading to long-lasting antitumor response. Such an experimental approach defines a new immunotherapeutic concept based on active modulation of the host immune system, promoting a shift from the prevailing immunosuppressive environment elicited by the tumor growth, to productive antitumor responses. This new immunotherapeutic concept is strongly supported by activating antigen-presenting cells (APCs) such as macrophages and dendritic cells (DCs), both systemically and inside tumor masses. Our group has pursued the concept of active immunotherapy procedures, based on the use of DNA vaccines or purified recombinant proteins combined with strong adjuvants, to induce antitumor protective responses against HPV-associated tumors. Both vaccine types are based on the fusion of HPV-16 E7 oncoprotein to the herpes simplex virus 1 (HSV-1) glycoprotein D (gD). Using the experimental model based on the subcutaneous transplantation of TC-1 cells, mice immunized with the vaccines elicited systemic E7-specific CD8^+^ T lymphocytes and controlled tumors at the initial growth stages 12,13. Here, we investigated a more potent treatment capable of controlling advanced-stage tumors using different HPV-associated tumor models.

## Methods

### Cell culture

The TC-1 tumor cell line was derived from the c-Ha-ras transformed C57BL/6 lung epithelium and the HPV-16 E6 and E7 oncogenes 14. The TC-1 LUC cell line was generated by the transfection of a retrovirus containing the luciferase gene, as previously described by Kim et al (2007) 15. Both cell lines were kindly provided by Dr. T.C. Wu (Johns Hopkins University, Baltimore, MD, USA). TC-1 and TC-1 LUC cells were cultured in RPMI medium (Thermo Fisher Scientific, Waltham, USA) containing 10% fetal bovine serum (Thermo Fisher Scientific, Waltham, USA) and G418 (Geneticin; Merck, Darmstadt, Germany) at a final concentration of 400 μg/mL and maintained at 37 °C and 5% CO_2_. On the day of tumor transplantation, cells were treated with trypsin, washed, and suspended in phosphate buffered saline (PBS) (pH 7.4) at an appropriate concentration for each experiment. Only those cultures with a cell viability equal to or greater than 90% by the Trypan Blue staining method were considered suitable for use.

### Mice and TC-1 tumor transplantation

All *in vivo* experiments were carried out with 6-8 week old C57BL/6 mice acquired from the animal facility unit of the Department of Pathology (Faculty of Veterinary Medicine and Zootechnics) at the University of Sao Paulo (USP, Sao Paulo, Brazil) and handled according to the norms established by the Institute of Biomedical Sciences (ICB/USP) Ethics Committee (protocol number 36 in 07/28/2014; protocol number 104 in 10/03/2017). To evaluate the therapeutic efficacy of cisplatin and immunotherapy, the mice received subcutaneous (SC) inoculation with 5x10^5^ TC-1 cells/100 μL in the right flank. Tumor growth was monitored and major (D) and minor (d) diameters were measured in mm with the aid of a pachymeter at least twice per week. Treatment was initiated when at least one of the diameters reached 10 mm, corresponding to an initial tumor volume (day 0) of 200-300 mm^3^, according to the formula (Dxd^2^)/2. Mice were euthanized when tumor volume reached 1,000 mm^3^, or when at least one of the tumor diameters reached 15 mm. The combination of cisplatin and immunotherapy was also evaluated in the intravaginal (IVAG) tumor model. In this case, TC-1 LUC cells were used to monitor tumor growth through the emission of luminescence. To coordinate the estrous cycle, tumor cell inoculation was preceded by SC administration of DepoProvera™ (medroxyprogesterone acetate; Pfizer, New York, USA) 5 days before TC-1 cells implantation at a concentration of 3 mg/mL. Tumor cells transplantation was performed by injecting 1x10^5^ TC-1 LUC cells/20 μL. From day 10 of TC-1 LUC cells transplantation, mice were monitored periodically by analyzing the luminescence emission in the IVIS Spectrum equipment (Perkin Elmer, Waltham, USA) at the Center for Research Support Facilities (CEFAP, ICB, USP). Mice were euthanized when the total flux reached 10^10^ photons/second.

### *In vivo* administration of cisplatin

Cis-Diamineplatinum (II) dichloride (Merck) was dissolved in sterile saline solution for 1 h at 60°C. The cisplatin solution was administered via the intravenous (IV) route through the retro-orbital plexus in amounts proportional to the body mass to obtain doses of 10 mg/kg. The body weight of mice was measured twice per week and values were presented in relation to the body mass (in grams, g), or as a change in body weight considering 100% as the weight value obtained on day 0.

### Mice immunization with pgDE7h DNA vaccine or gDE7 protein vaccine

Immunization by the intramuscular (IM) route with pgDE7h was performed in a total volume of 100 μL per dose, with 50 μL administered in each paw. Mice receiving electroporation were previously anesthetized intraperitoneally with a mixture of 75 mg/kg ketamine (Ceva Santé Animale, Libourne, France) and 10 mg/kg xylazine (Ceva Santé Animale, Libourne, France). For electroporation, the electrode CUY560-5-0.5 was used, consisting of a pair of parallel fixed needles 0.5 mm in diameter with a space of 5 mm between them. The electrode was inserted into the anterior tibial muscle shortly after inoculation of the vaccine. Six electric pulses of 130 V each were applied with a duration of 450 milliseconds. Electrical pulses were delivered using the NEPA21 SuperEletroporator equipment (NepaGene, Ichikawa City, Japan). The therapeutic gDE7-based protein vaccine (PTN) was administered following a regimen of two SC doses, at day 3 and 7 after cisplatin treatment. Each dose contained 30 μg of the gDE7 protein admixed with 50 μg of the poly(I:C) (pIC; InVivoGen, San Diego, USA) adjuvant, diluted in saline solution (total volume of 100 μL), and inoculated in the right rear flank of the mice.

### Intracellular IFN-γ staining

Peripheral blood cells were collected from the submandibular plexus of mice in tubes containing 30 μL of heparin (1 U/μL). The cells were treated for 5 min at 25°C with Ack Lising Buffer (Thermo Fisher Scientific, Waltham, USA) for red blood cell rupture, and centrifuged at 600 g for 5 min. The cells were incubated at a concentration of 10^6^ cells/well for 6 h at 37 ºC and 5% CO_2_ in the presence of 10 µg/mL Brefeldin A (GolgiPlug; BD Biosciences, San Jose, USA) and 5 ng/mL IL-2 (Sigma) in the absence or presence of the specific E7 peptide (amino acids 49-57; RAHYNIVTF; 300 ng/well) (GenScript, Piscataway, Nova Jersey, EUA) 16. After that period, the cells were incubated for 30 min at 4ºC with anti-CD8a antibody conjugated to fluorochrome BB515 (BD Biosciences) or APC (BD Biosciences). After permeabilization with the Cytofix/Cytoperm solution (BD Biosciences) for 10 min at 4ºC, cells were treated with anti-IFN-γ antibody conjugated to PE (BioLegend) or BV421 (BioLegend, San Diego, USA) for 30 min at 4ºC. The cells were then suspended in PBS and examined by flow cytometry. Samples were acquired on a LSR Fortessa flow cytometer (BD Biosciences) and analyzed using the FlowJo software (BD Biosciences). The percentage of CD8^+^ IFN-γ^+^ T cells over all CD8^+^ T cells was determined.

### Biochemical analysis of blood samples

Blood samples were collected three days after the end of the treatments from the submandibular plexus of each mice: on day 10 for "Naïve", "Untreated”, and "1CIS/DNA and 1CIS/PNT" groups, and on day 17 for the "3CIS" group. Serum was obtained after centrifugation at 2,600 g for 15 min at 4ºC, and used to obtain aspartate aminotransferase (AST) (Laborclin, Pinhais, Brazil), alanine aminotransferase (ALT) (Laborclin), blood urea nitrogen (BUN) (Wiener Lab Group, Sao Paulo, Brazil), and creatinine (Wiener lab) levels, according to the manufacturer's instructions.

### Analysis of glutathione (GSH) in kidney samples

Kidney samples were collected individually (one kidney per mice), frozen in liquid nitrogen, and stored at -80ºC until use. The tissue was mechanically dissociated, and homogenization was carried out with a 5% sulfosalicylic acid solution. Samples were then centrifuged at 18,000 g for 30 min at 4ºC and the supernatant used for the analysis of GSH. The ratio between total and oxidized glutathione (GSH/GSSG) was obtained by kinetic analysis using the colorimetric method of detecting DTNB (Ellman's reagent). For the test, the 38185 Quantification kit for oxidized and reduced glutathione (Merck) was used, following the manufacturer's instructions.

### Cell population analysis in tumors implanted subcutaneously

Tumor samples were collected individually, and cell suspension was obtained after mechanical dissociation and incubation with collagenase D (Roche, Basiléia, Swiss) at a concentration of 0.22 U/mL per sample at 37 ºC for 40 min. Then, samples were washed twice with PBS 1X (pH 7.4), filtered in a 70 µm cell strainer (BD Biosciences), and resuspended in solutions containing anti-CD45-PerCpCy5.5 (BioLegend), anti-CD11c-PE (BD Biosciences), MHC-II-BV421 (BioLegend), F4/80-FITC (Thermo Fischer Scientific), anti-CD86-APC (BioLegend), anti-CD11b-Alexa-Fluor488 (BioLegend), anti-Gr-1-PE (BD Biosciences), CD8a-BV605 (BioLegend), and anti-IFN-γ-PE (BioLegend) mAbs for 30 min at 4ºC. For analysis of E7-specific intratumoral CD8 T cells, samples were stained with the APC-labeled H-2Db E7-specific dextramer (Immudex, Copenhage, Denmark), and subsequently stained with anti-CD8a-Pacific Blue (BioLegend). After two washes, cells were resuspended in PBS, acquired in a LSR Fortessa flow cytometer (BD Biosciences), and analyzed using the Flow Jo software (BD Biosciences).

### TC-1 cell re-challenge models

To evaluate the antitumor memory responses and simulate tumor relapse events, tumor-free mice were reinjected with TC-1 cells 90 days after the first tumor implantation. Mice that showed complete regression of SC tumors were re-challenged with 5x10^6^ TC-1/100 μL cells in the right flank. Mice that showed complete regression of intravaginal tumors were re-challenged at the same region injecting 1x10^6^ TC-1 LUC/20 μL, or in the tongue with 1x10^6^ TC-1 LUC/10 μL cells. The group re-challenged in the tongue received another implantation 60 days later with 1x10^6^ TC-1 LUC/10 μL cells. The luminescence emission was acquired using the IVIS Spectrum equipment (Perkin Elmer) at the Center for Research Support Facilities (CEFAP, ICB, USP).

### Analysis of the T cell memory response in spleen samples

The cells were incubated at a concentration of 3x10^6^ cells/well for 6 h at 37 ºC and 5% CO_2_ with 10 µg/mL Brefeldin A (GolgiPlug; BD Biosciences), 5 ng/mL IL-2 (Thermo Fisher Scientific), and 300 ng/well of E7 peptide^16^ (amino acids 49-57; RAHYNIVTF; GenScript). After incubation, cells were stained for 30 min at 4 ºC with anti-CD8a-APC (BioLegend), anti-CD44-FITC (BioLegend), anti-CD62L-BV421 (BioLegend), anti-KLRG1-PE (BioLegend), and anti-CD127-PECy7 (BD Biosciences) antibodies. After fixation/permeabilization with the Cytofix/Cytoperm solution (BD Biosciences) for 10 min at 4 ºC, cells were stained with anti-IFN-γ antibody conjugated to Alexa700 (BD Biosciences) for 30 min at 4 ºC. The cells were then resuspended in PBS and examined by flow cytometry using the LSR Fortessa device (BD Biosciences).

### Statistical analysis

Statistical analyses were performed using Prism software (GraphPad, San Diego, USA). Analysis was performed using one-way or two-way analysis of variance (ANOVA), and the results were confirmed through multiple comparisons by Bonferroni's test. The unpaired *t*-test was used for comparison between two groups. Survival curves were analyzed by the log-rank (Mantel-Cox) test. Values of *p* <0.05 were considered significant.

## Results

### Combination of cisplatin and gD-based vaccines results in synergistic control of HPV-associated advanced-stage tumors with reduced toxic effects

**Figure 1:**
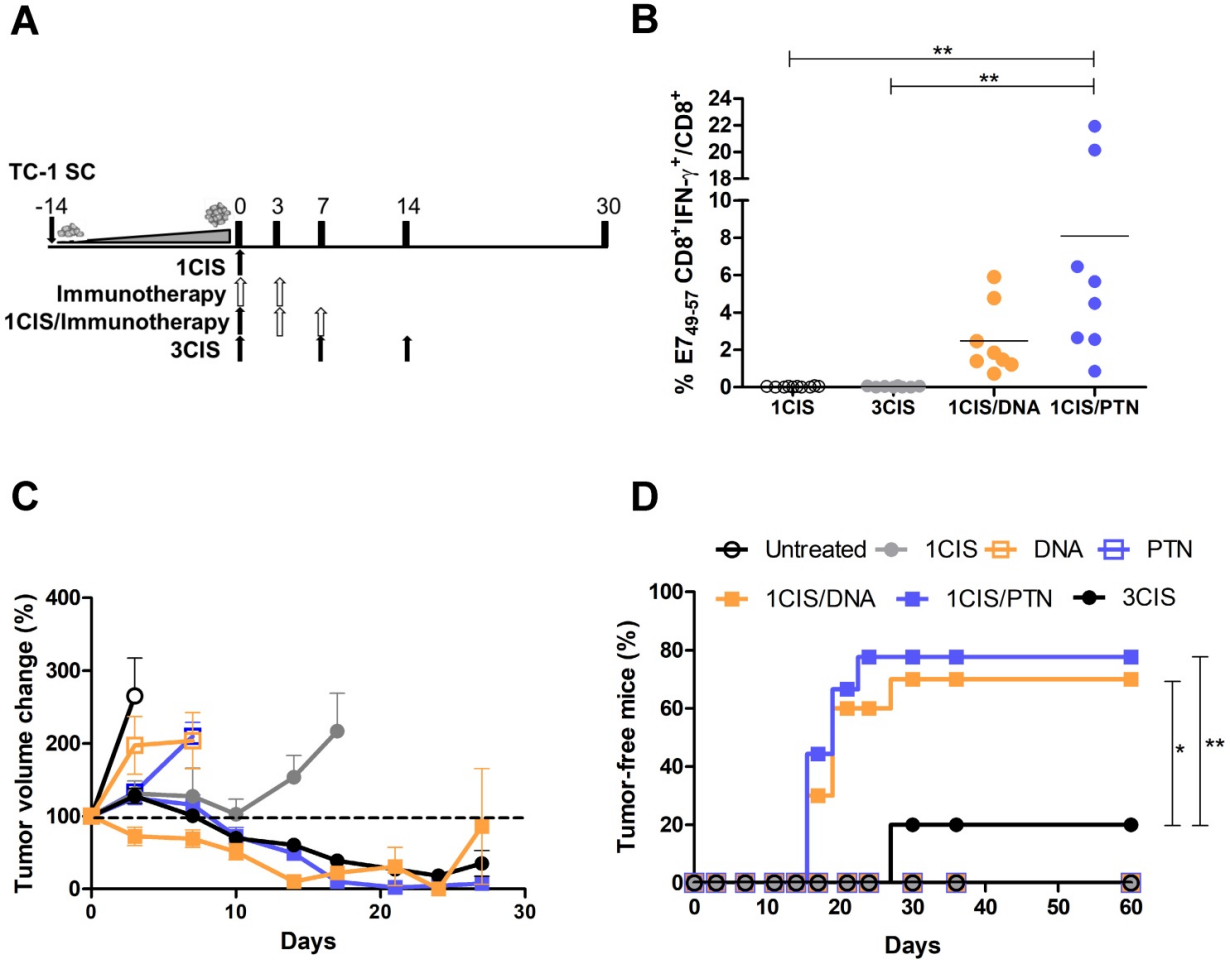
** Combination of cisplatin and pgDE7h or purified gDE7 enhances therapeutic antitumor responses. (A)** Tumor-bearing C57BL/6 mice were treated with one or three doses of cisplatin, or immunotherapy (DNA meaning immunization with pgDE7h delivered by electroporation, and PTN meaning immunization with gDE7 mixed with poly (I:C)), or the combination of both treatments (one dose of cisplatin combined with DNA or PTN). **(B)** Frequency of systemic CD8^+^IFN-γ^+^/CD8^+^ T cells after *in vitro* stimulation with the CD8^+^ T cell-specific E7 peptide (^49^RAHYNIVTF^57^) at day 14. **(C)** Tumor volume change of DNA- and protein-based treated mice compared to day 0 (~10 mm diameter tumors). **(D)** Percentage of mice that showed complete tumor regression after treatment during the follow-up period. Data represent the mean +SD of 8-10 mice per group from two independently performed experiments. Statistical significance: (*) *P* < 0.05, (**) *P* < 0.01, as determined by one-way ANOVA followed by *Bonferroni post hoc* analysis and log-rank (*Mantel-Cox*) test for tumor-free data (survival curve).

We previously reported the antitumor effects of pgDE7h and gDE7 vaccines. Optimal results were achieved when pgDE7h was delivered by *in vivo* electroporation 17, and when gDE7 was combined with the adjuvant pIC 18. To improve the performance of both vaccines in advanced-stage tumors, we combined the active immunotherapies with cisplatin. For that purpose, mice were first treated with cisplatin when tumors reached 10 mm in diameter (day 0) (Fig. 1A). No systemic E7-specific CD8^+^ T cells were detected in mice treated with three doses of cisplatin (Fig. 1B), but showed transient control of tumor growth (Fig. 1C) and 20% of antitumor protection (Fig. 1D). In contrast, the combination of cisplatin with the DNA or protein-based vaccines promoted a significant increase in circulating E7-specific CD8^+^ T cells (Fig. 1B ), led to enhanced tumor control (Fig. 1C; Fig. S1) and complete tumor regression in 70% or 80% of the immunized mice, respectively (Fig. 1D). Under the tested conditions, mice treated with two doses of either DNA or protein-based vaccines alone, or one dose of cisplatin failed to induce tumor growth control (Fig. 1C-D). In addition, the administration of two doses of cisplatin did not control the tumor burden (Fig. S2A-C) and exhibited severe toxicity, as demonstrated by significant body-weight loss (Fig. S2D). Remarkably, the synergistic effects of cisplatin required administration of pgDE7h by electroporation or the co-administration of gDE7 and pIC (Fig. S3). Collectively, our data demonstrate that the combination of cisplatin with either DNA or protein-based vaccines led to synergistic effects regarding the effective control of advanced-stage tumors.

**Figure 2:**
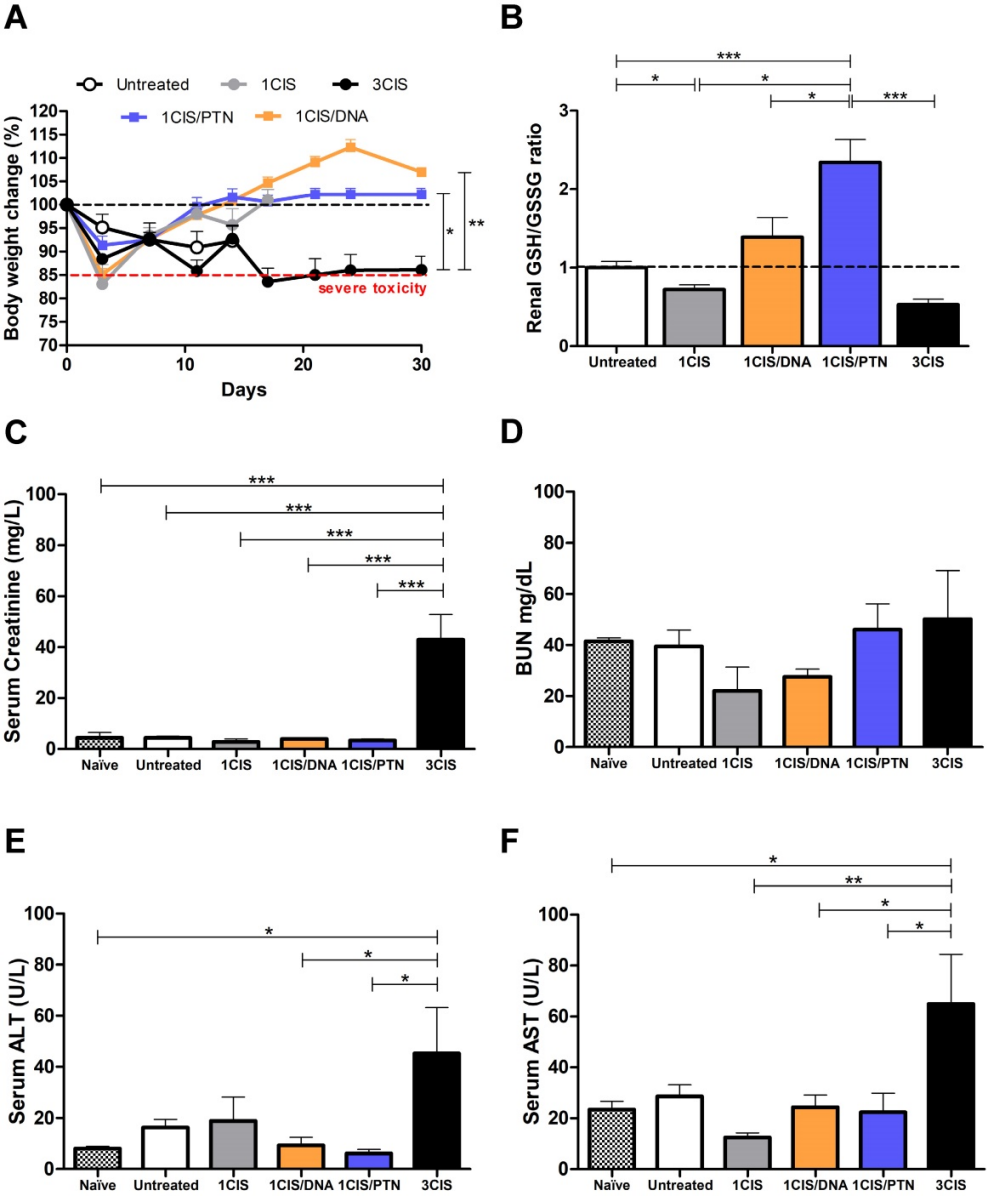
** Combination of cisplatin and immunotherapy (pgDE7h or gDE7**) **reduces toxicity of anti-tumor treatment.** Tumor-bearing C57BL/6 mice were treated with cisplatin, pgDE7h and electroporation, or gDE7 mixed with poly(I:C), or the combined chemotherapy/immunotherapy treatment, as presented in Fig. 1.** (A)** Percentage of weight loss throughout the treatment protocol (relative to initial weight on day 0). Non-treated group (n=5), other groups (n=8-10). **(B)** Kidney redox status expressed as GSH/GSSG ratio assessed three days after each treatment. Serum concentration of (**C**) creatinine, **(D)** blood urea nitrogen, BUN, **(E)** alanine aminotransferase, ALT, and **(F)** aspartate aminotransferase, AST, obtained 3 days after each treatment. Data represent the mean +SD of 5-8 mice per group from two independent experiments. Statistical significance: (*) *P* < 0.05, (**) *P* < 0.01, (***) *P* < 0.001, as determined by one-way ANOVA followed by *Bonferroni post hoc* analysis.

**Figure 3:**
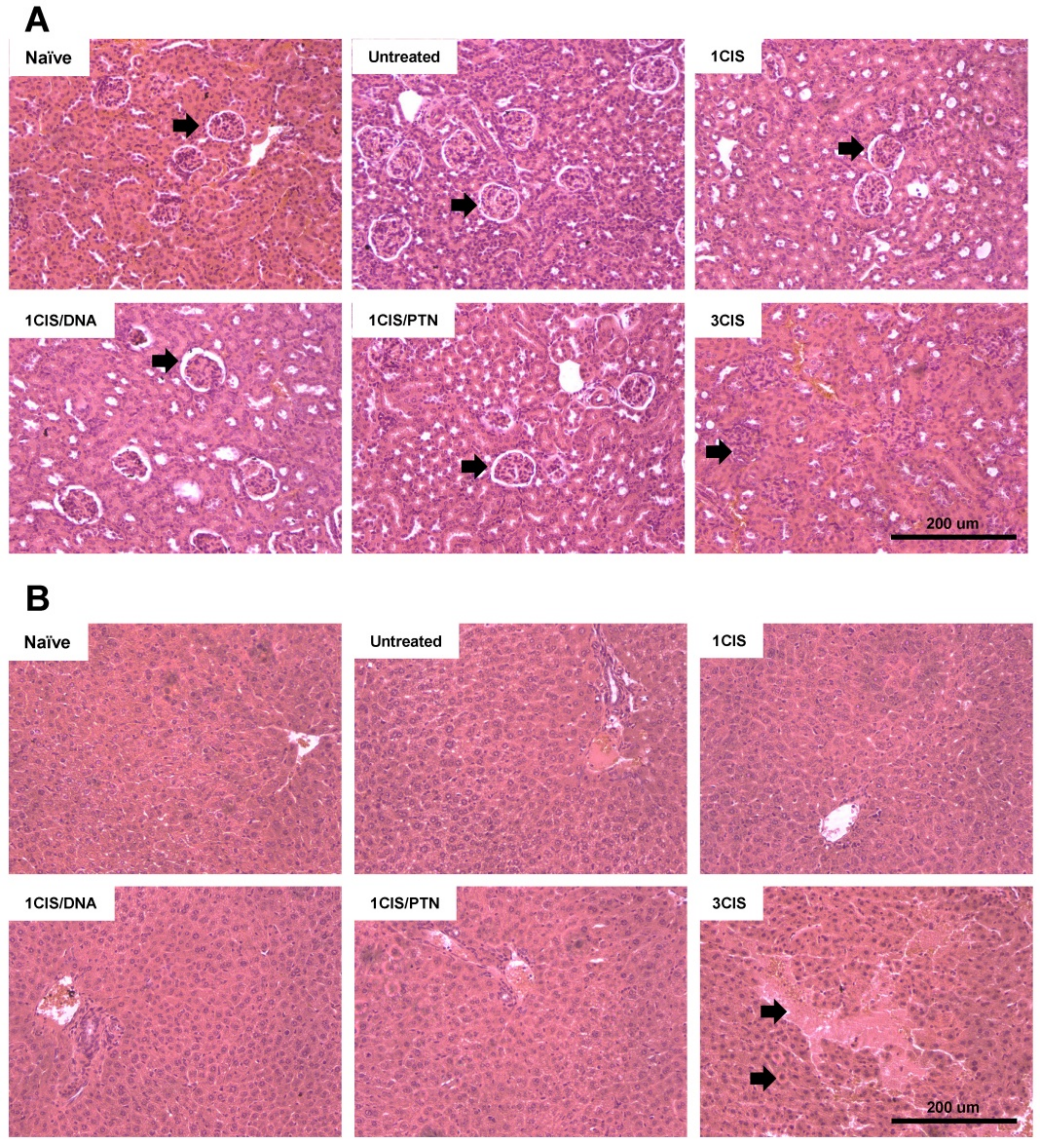
** The combined treatment induces less histological damages than repeated doses of cisplatin.** Tumor-bearing C57BL/6 mice were treated with cisplatin or the combined chemotherapy/immunotherapy treatment as previously described. **(A)** Hematoxylin and eosin (H&E) stained sections of a representative kidney tissue three days post treatment (n=3; 20x magnification). **(B)** H&E stained sections of a representative liver tissue three days post treatment (n=3; 20x magnification). Histological findings (indicated by arrows) are depicted in Table S1.

We also investigated the acute systemic toxicity associated with the use of cisplatin and the combined chemo/immunotherapy. As demonstrated in Fig. 2A, three doses of cisplatin induced severe toxicity in mice leading to sustained 15% body weight loss (day 14). In contrast, the combination of one cisplatin dose with two vaccine doses, either DNA or purified protein, caused an initial moderate weight loss that was quickly reverted (Fig. 2A). Mice submitted only to the immunotherapy (DNA or protein) showed no significant weight loss concerning sham-treated mice (data not shown). In addition, increased glutathione (GSH/GSSG) levels, indicative of reduced kidney damage, were detected in mice treated with the combined chemo/immunotherapy regimen, especially in mice administered with the protein-based vaccine (Fig. 2B). Conversely, mice treated with three cisplatin doses showed high serum levels of creatinine, ALT, and AST, indicative of kidney and liver damage. In contrast, treatment with one dose of cisplatin and two vaccine doses did not show any significant increase in the serum levels of these tissue damage markers (Fig. 2C, E, and F). No differences in BUN levels were found among all experimental groups (Fig. 2D). To confirm the toxicity induced by cisplatin, we also assessed tissue damage by histological analyses of kidney and liver of mice submitted to the different tested treatments (Fig. 3). The combined treatment induced fewer histological changes than repeated cisplatin doses in both tissues. While repeated doses of cisplatin have induced capillary thrombosis and ischemic changes in the kidneys of treated mice, the combination of a single dose of cisplatin with immunotherapy induced only mild glomerular congestion. Similarly, repeated doses of cisplatin led to reactive changes in hepatocytes and intraparenchymal hemorrhage while the chemo/immunotherapy protocol preserved the liver tissue (Table S1). Thus, we concluded that the combination of one dose of cisplatin with either pgDE7h or gDE7 allowed the reduction of cisplatin toxic effects without affecting the antitumor responses.

### Combined use of cisplatin and gDE7 induces tumor infiltration of immunomodulatory cell subsets and E7-specific CD8^+^ T cells, and reduces the frequency of myeloid suppressor cells

**Figure 4:**
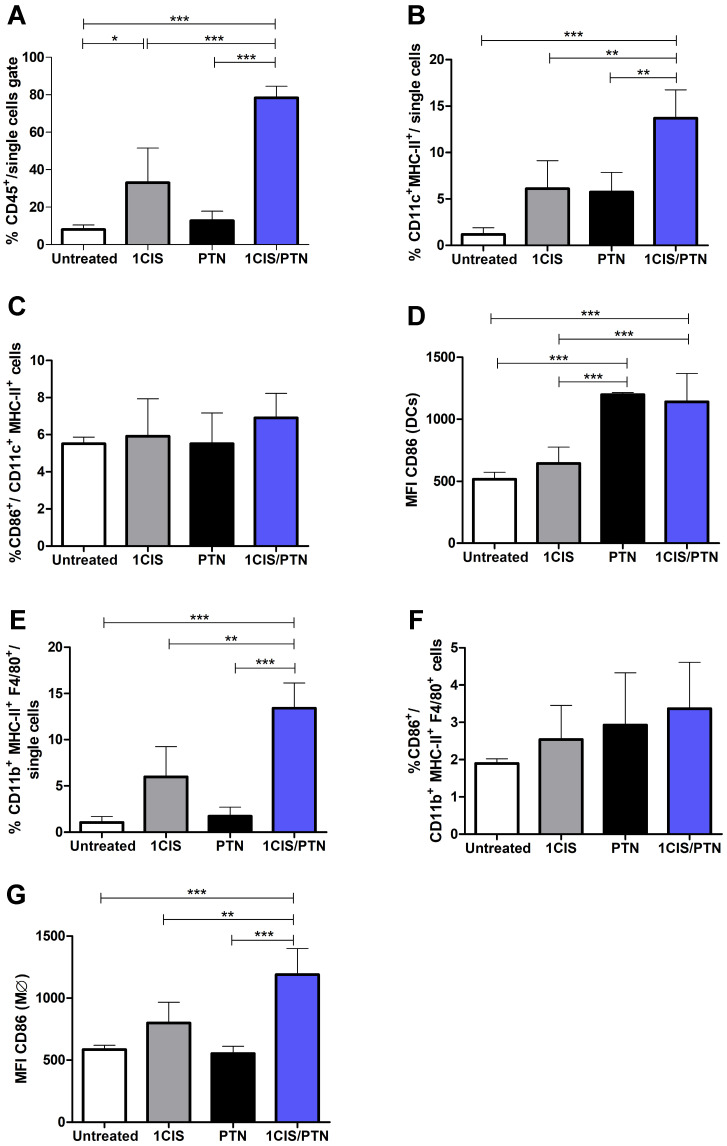
** Immunization with purified gDE7 combined with cisplatin enhances tumor infiltration of antigen-presenting cells.** Tumor-bearing C57BL/6 mice were treated with one dose of cisplatin, gDE7 admixed with poly (I:C) or cisplatin combined with gDE7 admixed with poly(I:C). Tumor samples were collected at day 10 and analyzed by flow cytometry. Frequency of **(A)** CD45^+^ infiltrating cells, **(B)** CD11c^+^MHCII^+^ dendritic cells (DCs) and **(C)** CD11c^+^MHCII^+^CD86^+^ activated DCs**. (D)** Median Fluorescence Intensity (MFI) of CD86 on CD11c^+^MHCII^+^ DCs. **(E)** Percentage of CD11b^+^MHCII^+^F4/80^+^ macrophages and **(F)** CD11b^+^MHCII^+^F4/80^+^CD86^+^ activated macrophages. **(G)** MFI of CD86 on CD11b^+^MHCII^+^F4/80^+^ macrophages. Gate strategies for detection of tumor-infiltrating antigen presenting cells are depicted in Figure S4. Data are expressed as the mean + SD from one of two independent experiments with similar results (n=3-5). (**) *P* < 0.01, (***) *P* < 0.001, as determined by one-way ANOVA followed by *Bonferroni post hoc* analysis.

Further testing of the benefits and potential synergistic effects associated with the combination of cisplatin and gD-based vaccines focused on the induced immune responses in the tumor microenvironment. For these experiments, mice received one dose of cisplatin, the protein-based vaccine alone, or the combined treatment. Under the tested experimental conditions, mice treated with cisplatin combined with purified gDE7 admixed with pIC showed higher frequencies of intratumoral CD45^+^ cells (Fig. 4A) and CD11c^+^MHC-II^+^ DCs, compared to mice treated with cisplatin or the protein-based vaccine alone (Fig. 4B). Although we did not find differences in the frequency of DCs that express CD86 (Fig. 4C), DCs from mice treated with the chemo/immunotherapy or the protein-based vaccine alone showed higher expression of this costimulatory molecule, which correlate with a greater immune-stimulatory capacity (Fig. 4D). Similarly, mice treated with the combination of cisplatin and gDE7 admixed with pIC elicited increased frequency of CD11b^+^F4/80^+^MHCII^+^ macrophages compared to mice treated with cisplatin, protein-based vaccine alone or untreated mice (Fig. 4E). Besides that, the combined treatment did not increase the frequency of CD86^+^ intratumoral macrophages, but, otherwise, induced a higher CD86 expression in these cells, compared to mice submitted to the isolated treatments (Fig. 4F-G). The gating strategy applied in the analyses of these cell subsets is shown in Fig. S4.

**Figure 5:**
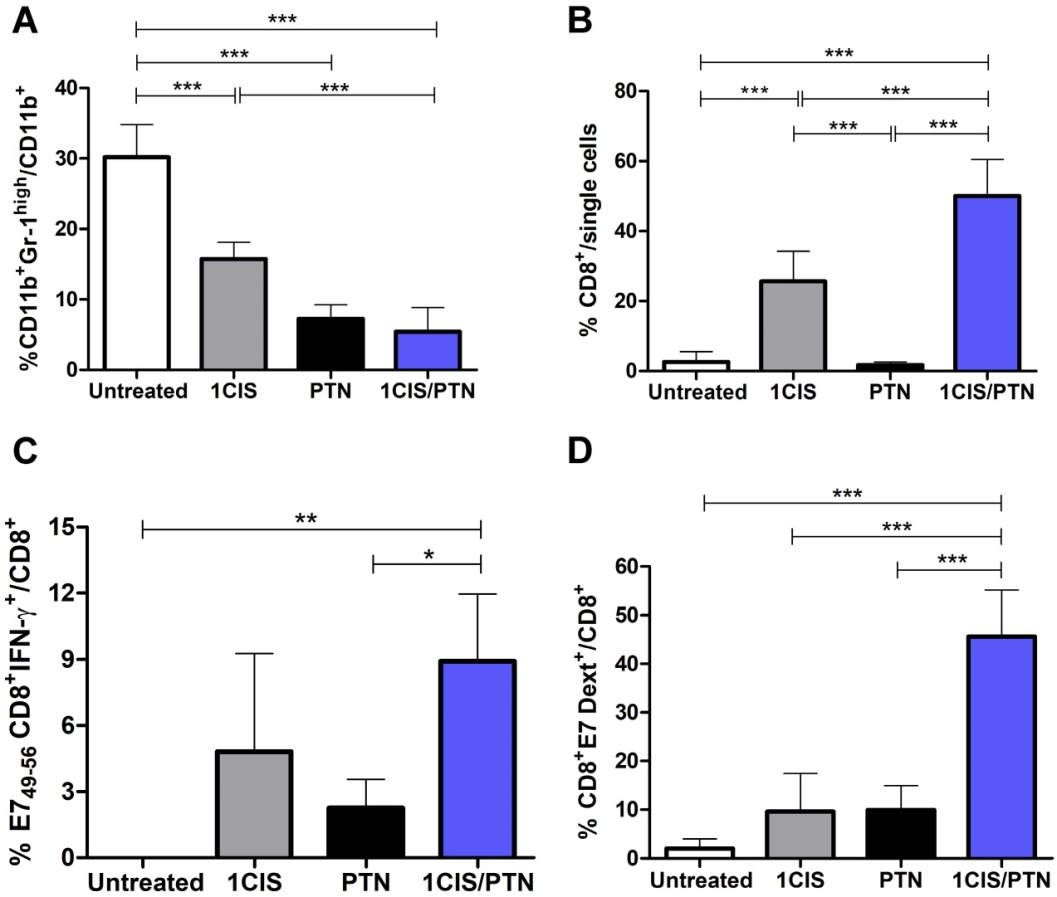
** Administration of purified gDE7 and poly(I:C) combined with cisplatin reduces the frequency of myeloid suppressor cells and enhances tumor infiltration of E7-specific CD8^+^ T cells.** Tumor-bearing C57BL/6 mice were treated with cisplatin, gDE7 admixed with poly (I:C) or the combined chemo/immunotherapy protocol. Tumor samples were obtained at day 10 and analyzed by flow cytometry. Frequency of **(A)** myeloid-derived suppressor cells (MDSCs) represented by CD11b^+^Gr-1^high^ cells, **(B)** CD8^+^ T cells, **(C)** IFN-γ^+^ CD8^+^ T cells after *in vitro* stimulation with the CD8-specific E7 peptide (^49^RAHYNIVTF^57^), and **(D)** E7-specific CD8^+^ T cells labeled with the E7 H-2Db-specific dextramer. Data are expressed as the mean +SD of one of two independent experiments with similar results (n=5-6). (*) *P* < 0.05, (**) P < 0.01, (***) *P* < 0.001, as determined by one-way ANOVA followed by *Bonferroni post hoc* analysis.

In addition, we evaluated the frequency of CD11b^+^Gr-1^high^ myeloid-derived suppressor cells (MDSCs) in mice submitted to the tested treatments. Our results revealed lower frequencies of intratumoral MDSCs in mice treated with cisplatin or protein-based vaccine alone, which was further reduced when cisplatin was combined with gDE7 admixed with pIC immunization (Fig. 5A). Treatment with cisplatin combined with the protein vaccine induced a significant increase in tumor-infiltrating CD8^+^ T cells (Fig. 5B), particularly antigen-specific CD8^+^ T cells, compared with mice treated with the protein-based vaccine alone (Fig. 5C). Antigen-specific CD8^+^ T cells, denoted Dex^+^CD8^+^E7 T cells, represented 40% of the intratumoral CD8^+^ T cell population in mice submitted to the chemo/immunotherapy (Fig. 5D). Altogether, these data demonstrate that administration of cisplatin and vaccination with gDE7 synergistically increases the frequency of tumor-infiltrating dendritic cells, macrophages, and antigen-specific CD8^+^ T cells, and reduces the frequency of intratumoral immunosuppressive cells.

### Combination of cisplatin and gD-based vaccines induces synergistic therapeutic effects in advanced tumors at different anatomical sites

**Figure 6:**
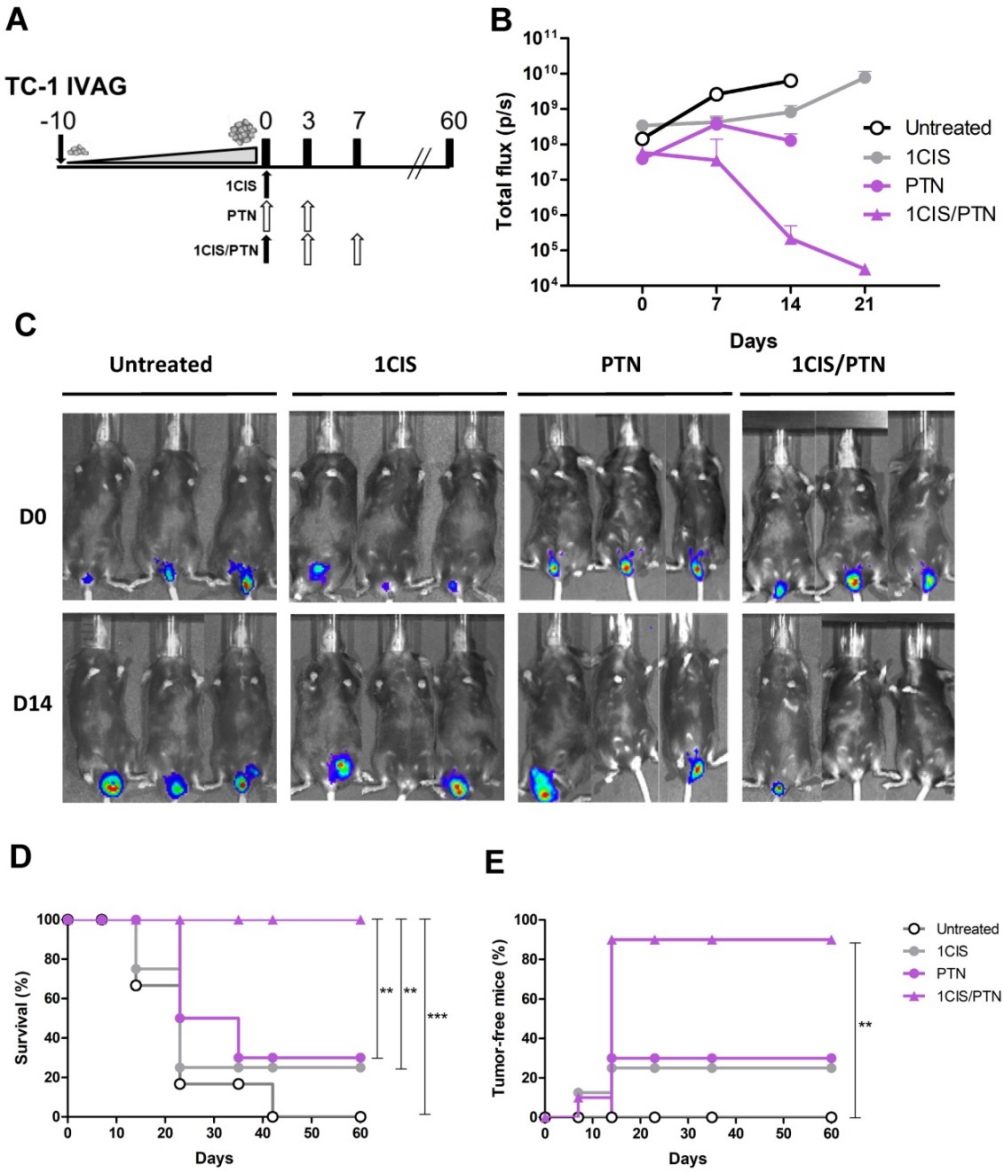
** Combined treatment based on purified gDE7 admixed with poly(I:C) and cisplatin confers enhanced protection to intravaginal tumors. (A)** Treatment representative sketch. C57BL/6 mice were inoculated with 10^5^ TC-1 LUC cells intravaginally and treated with one dose of cisplatin, gDE7 admixed with poly(I:C) or with the combined chemo/immunotherapy. Tumor growth was evaluated by bioluminescence emission. **(B)** Bioluminescence emissions from tumors measured as individual values of photons per second (p/s). **(C)** Representative bioluminescence images of vaginal TC-1 LUC tumors until day 14. **(D)** Percentage of survival, and **(E)** tumor-free mice followed until 60 days after the initial treatment. Data are expressed as the mean ± SD of two independent combined experiments (n=8-10). Statistical significance: (**) *P* < 0.01, (***) *P* < 0.001, as determined by log-rank (*Mantel-Cox*) test.

Tumors induced by HPV are found at different mucosal epithelia and may show distinct tumor microenvironments and immune surveillance mechanisms. To evaluate the therapeutic performance of the combined chemo/immunotherapy, we tested the treatment in an orthotopic tumor model after transplantation of TC-1 LUC cells into the vagina of the C57BL/6 mice, as an approach to simulate conditions found in human cervical and vaginal cancers. Tumor-bearing mice were first treated with a single cisplatin dose, followed by administration of two doses of the protein vaccine 10 days after the implantation of TC-1 LUC cells (Fig 6A), when tumor bioluminescence was approximately 10^8^ photons/second (Fig 6B). The results showed that administration of cisplatin or the protein-based vaccine alone induced partial control of tumor growth, leading to tumor regression in 25% and 30% of the mice, respectively (Fig 6C-D). Conversely, the combined treatment induced complete tumor regression in 90% of treated mice and 100% of survival during the observation period (Fig. 6D-E). Similar results were also observed in mice treated with cisplatin and the DNA vaccine pgDE7h (Fig. S5). Collectively, these data confirm the synergistic effects of the combined chemo/immunotherapy in HPV-associated tumors at the vaginal epithelium.

### Chemo/immunotherapy induces immunological memory responses and confers long-term protection from tumor relapses in different anatomical sites

**Figure 7:**
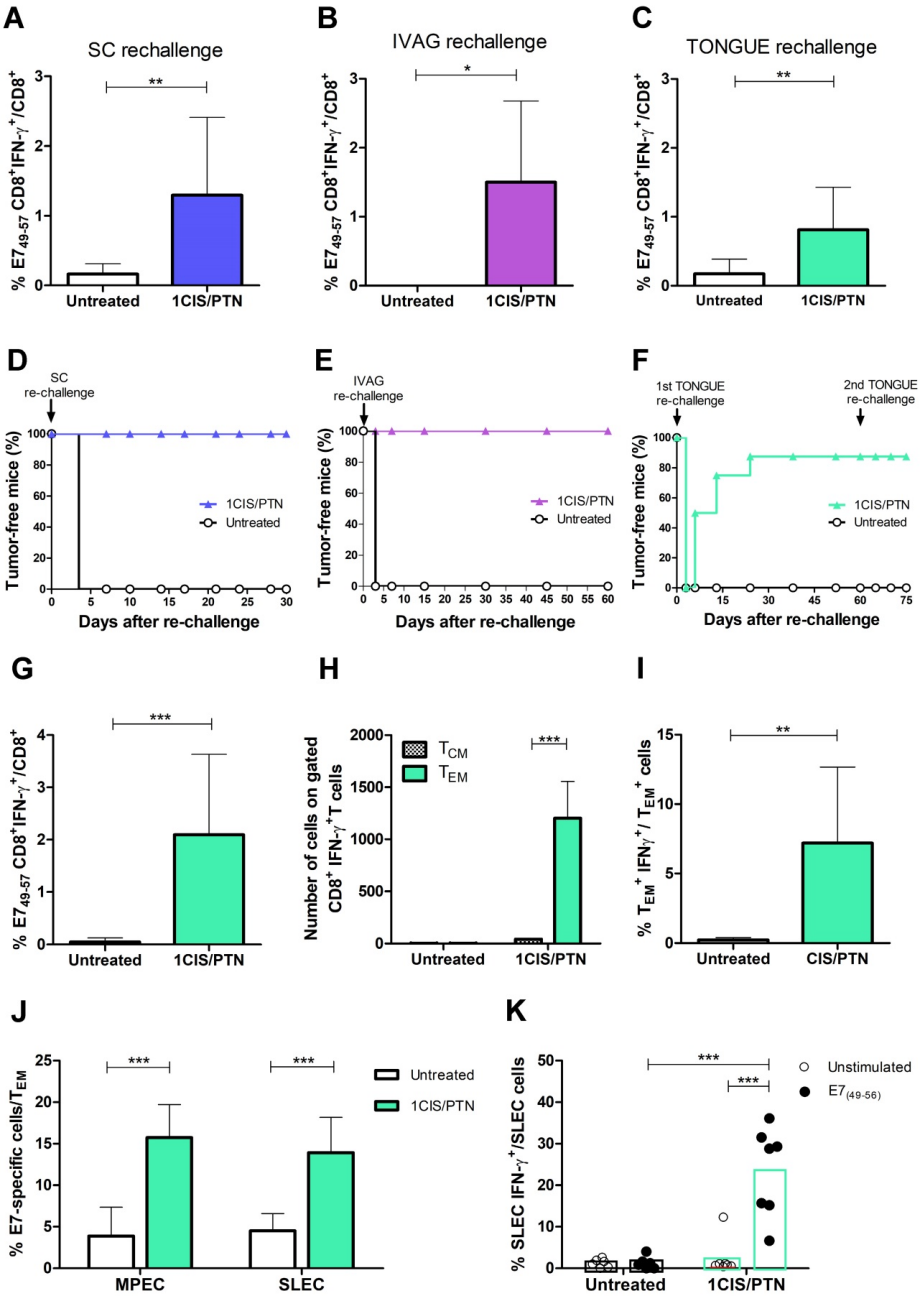
** Combination of cisplatin and gDE7 admixed to poly(I:C) enhances the frequency of systemic tumor-specific CD8^+^ T cells and prevents tumor relapses in different anatomical sites.** C57BL/6 mice were inoculated with 10^5^ TC-1 cells and subsequently treated with the combined chemo/immunotherapy. At day 60, tumor-free mice were rechallenged to simulate tumor relapses. Frequency of CD8^+^IFN-γ^+^/CD8^+^ T cells after *in vitro* stimulation with the CD8-specific E7 peptide (^49^RAHYNIVTF^57^) was determined seven days after: **(A)** subcutaneous, **(B)** intravaginal, and **(C)** intratongue reinoculation of 10^6^ TC-1 cells. Percentages of tumor-free mice after **(D)** subcutaneous, **(E)** intravaginal, or **(F)** intratongue reinoculation of TC-1 cells. Tumor-free mice of tongue rechallenge were subjected to a second tumor rechallenge with 10^6^ TC-1 cells at the same anatomical site. Spleens were collected 14 days after the second rechallenge and analyzed by flow cytometry. **(G)** Frequency of IFN-γ^+^CD8^+^ T cells after *in vitro* stimulation with the CD8-specific E7 peptide (^49^RAHYNIVTF^57^). **(H)** Frequency of CD8^+^CD44^+^CD62L^-^ (T_EM_), and CD8^+^CD44^+^CD62L^+^ (T_CM_) gated on CD8^+^IFN-γ^+^ T cells, and **(I)** frequency of IFN-γ^+^-expressing T_EM_ cells. **(J)** Frequency of CD127^-^KLRG1^+^ T_EM_ (SLEC) and CD127^+^KLRG1^-^ T_EM_ (MPEC) splenic cells, and **(K)** IFN-γ^+^-expressing SLEC T_EM_ cells obtained after *in vitro* stimulation with or without the CD8^+^ T cell-specific E7 peptide (^49^RAHYNIVTF^57^) from individual spleen samples. Data are expressed as the mean +SD of two independently performed experiments (n=8-10). Statistical significance: (*) *P* < 0.05, (**) *P* < 0.01, (***) *P* < 0.001, as determined by two-way ANOVA or *t*-test.

A key feature distinguishing passive immunotherapy approaches from those based on active immunotherapies (vaccines) is the induction of immunological memory to cancer-associated antigens. To examine the induction of immunological memory responses, mice were evaluated for long-term antitumor protection including the capacity of preventing tumor recurrence in different anatomical sites. For that purpose, mice bearing subcutaneous or intravaginal tumors were initially administered with the chemo/immunotherapeutic regimen. Tumor-free mice were subsequently re-challenged with a 10-fold higher load of TC-1 cells at the same anatomical site 60 days after the initial treatment. All mice administered with the combined treatment and re-challenged with TC-1 cells had induced high frequencies of circulating E7-specific IFN-γ-producing CD8^+^ T cells (Fig. 7A-B) and did not develop tumors (Fig. 7D-E).

We also evaluated if long-term tumor protection would also be detected at an anatomical site different from the initial site used to inoculate the TC-1 cells. For that purpose, mice with complete regression of primary intravaginal tumors were re-challenged with a 10-fold higher load of TC-1 LUC cells in the tongue. In these conditions, tumor re-challenged mice, at an anatomical site different from the primary tumor, showed enhanced frequencies of circulating E7-specific IFN-γ-producing CD8^+^ T cells (Fig. 7C) and 90% of tumor rejection, remaining tumor-free until the end of the follow-up period (Fig.7F). Surviving mice were administered a second TC-1 LUC cells transplantation in the tongue two months later to assess the induced cellular responses. Spleen cells were collected two weeks after the second tumor cell challenge and tested for the presence of splenic antigen-specific CD8^+^ T cells (Fig. 7G), effector and central memory CD8^+^ T cells (Fig. 7H-I), and memory precursors (MPEC) and short-lived (SLEC) effector cells (Fig. 7J-K). Higher frequencies of MPEC, SLEC, and IFN-g^+^ cells were detected among E7-specific effector CD8^+^ T cells in mice treated with chemo/immunotherapy. Collectively, our data show that cisplatin combined with gDE7 immunization led to long-term antitumor protection in mice. More relevantly, mice administered with the combined chemo/immunotherapy regimen were capable of preventing tumor relapses, experimentally simulated by re-challenge with TC-1 cells at different epithelial sites. The long-term antitumor protection correlated with the activation of effector memory CD8^+^ T cells capable of migrating to peripheral tissues and responding to tumor cells.

## Discussion

In this study, we demonstrated that combining active immunotherapy against HPV-associated tumors with cisplatin induces synergistic effects, promoting enhanced eradication of tumors at advanced growth stages and prevent tumor relapses with lower toxicity. Previous work from our group showed that the DNA or protein-based therapeutic vaccines tested here, comprising HPV-16 E7 genetically fused to HSV-1 gD, led to enhanced activation of cytotoxic CD8^++^ T cells and therapeutic effect in tumors during the initial stage of development 12,13,18,19. Combining immunotherapy with chemotherapy resulted in increased antitumor immune responses, involving activation of intratumoral antigen-specific CD8^++^ T cells and APCs, with reduced frequencies of MDSCs. Importantly, the combined treatment generated more effective tumor protection at different anatomical sites, preventing tumor recurrence in subcutaneous, intravaginal, and tongue epithelia.

In humans, cervical cancer is the second most common cancer in Latin America and its mortality is projected to increase by 45% by 2030 despite HPV vaccination and screening efforts 20. In general, patients with advanced-stage and metastatic disease continue to have a poor prognosis with response rates ranging from 35-50% with current therapeutic options 21. The main goal of this work was to obtain an effective therapeutic protocol using active immunization that was able to eradicate tumors in an advanced growth stage. For this, we devised a treatment regimen that was initiated when the tumors reached diameters considered close to the limit of the preclinical model of tumors induced by HPV. In this protocol, we observed a slight control of tumor growth when using only protein or DNA-based vaccines; this was also observed in the group treated with only one dose of cisplatin. These results are better evidenced by observing the individual tumor growth curves for the non-combined treatments (Fig S1.), suggesting the potential of active immunization as a form of antitumor intervention. Of note, the inability of immunotherapy alone to reduce the tumor mass may be due to immunosuppression and difficulties in the infiltration of circulating cells in the tumor microenvironment, in addition to the increased proliferation. The combination with cisplatin, in turn, resulted in complete regression of tumors initially measuring 10 mm in diameter in almost 80% of mice treated with chemotherapy combined with the gDE7 vaccine. Similar studies have already been published with promising data on the combination of cisplatin with different immunotherapies against subcutaneous HPV-induced tumors. However, complete tumor regression occurred in non-established or only palpable tumors 22-26.

The benefits of this combination also include the reduction of at least one-third of the chemotherapy dose, and consequently, the elimination of deleterious effects triggered by weekly cisplatin doses. Cisplatin (cis-diamminedichloroplatinum or CDDP) is currently one of the most important cytostatic agents in treating a wide range of solid tumors, but the clinical usefulness of this drug is limited by the development of nephrotoxicity and hepatotoxicity, side-effects that may be produced in various animal models 27,28. Alterations in kidney functions are characterized by signs of injury, such as changes in creatinine clearance and GSH status. GSH is the most important intracellular endogenous thiol used as a free radical scavenger to clear free radicals induced by CDDP, which maintains cell integrity by avoiding oxidative damage. Elevated GSH plays an important role in protecting CDDP-induced toxicity 29. Our results revealed reduced GSH levels in mice treated with three doses of cisplatin. Nonetheless, enhanced levels of GSH in kidneys were shown in mice treated with cisplatin combined with both protein and DNA vaccines, which was not observed in the group treated with cisplatin alone (Fig. 2B). These data suggest protection from renal injury mainly using the protein vaccine, and the possible causes of this effect could be a subject of future investigations.

Although cisplatin is not considered a classical agent of immunogenic cell death, it induces HMGB1 release of tumor cells as shown previously, inducing the upregulation of costimulatory molecules on APCs intratumorally 22,30. The recruitment of APCs also stimulates the uptake, processing, and presentation of dead cell-associated antigens, eventually resulting in the priming of an adaptive immune response 31. In our work, we also demonstrated the capacity of combined treatment to enhance the CD86 expression in CD11c^+^MHCII^+^ dendritic cells and CD11b^+^MHCII^+^F4/80^+^ macrophages into the tumor, accompanied by a decrease in the number of CD11b^+^Gr-1^hi^ MDSCs. Several studies have already demonstrated the central role of CD8^+^ T cells in HPV-induced tumor control, either preclinical or clinical settings 32-36. In general, no changes in absolute T cell count or substantial alterations in CD4^+^, CD8^+^, or regulatory T cells phenotype were observed, but enhanced IFN-γ-producing CD8^+^ T cell response was achieved after *in vitro* stimulation of E6/E7-specific peptides. Here, we showed high frequencies of IFN-γ-producing CD8^+^ T cell reaching almost half the percentage of E7-specific lymphocytes within the total CD8^+^ T cell population in the tumor. As expected, most mice treated with cisplatin combined with either DNA or protein-based immunotherapies showed a complete tumor remission, even after subsequent TC-1 cells transplantations, indicating the potential of the combined treatment to induce a memory response.

Previous studies have found an increased risk of HPV-associated second primary cancers (SPCs), especially oropharyngeal HPV-SPCs, among cervical cancer survivors 37-40. Meanwhile, developed immunotherapeutic approaches targeting HPV oncogenes were very effective in inducing regression of established tumors in animal models, but the results strongly contrasted the poor clinical outcome to date. One possible cause of this discrepancy is represented by the non-orthotopic localization of the experimental tumor 41. Although many HPV-induced cancers are located in mucosal sites, most cancer immunotherapies were tested against SC tumors in preclinical settings 42. In this context, the submucosal administration of HPV-16 E7-expressing tumor cells in the cervicovaginal tract was previously described 43,44 and represents a useful way to mitigate the discrepancy between preclinical and clinical outcomes. Here, we showed the synergistic phenomenon between cisplatin and our therapeutic vaccines after implantation of mucosal tumors using the same parenteral vaccination regimen. For both protein and DNA-based vaccines, we observed 100% survival and 90% tumor regression in mice primarily carrying intravaginal tumors. In addition, all cured mice rejected a second tumor implantation in the intravaginal site. Interestingly, we evaluated the capacity of cisplatin combined with protein-based immunotherapy to prevent tumor recurrences after subsequent tumor cell transplantations in the tongue of mice that had been previously treated for an intravaginal tumor. Our results showed that 90% of mice clearly rejected the secondary developing tumor. This protection was accompanied by the enrichment of E7-specific CD8^+^ T cells systemically, particularly effector memory T cell subsets, which play an important role in antitumor immunosurveillance.

## Conclusions

The present work demonstrated a potent treatment for HPV-associated tumors involving cisplatin and two active immunotherapeutic strategies based on either DNA or protein-based vaccines. The observed outcomes were a consequence of the induction of an effector memory T cell response, both locally and systemically, as demonstrated by our rechallenge experiments. This study presents relevant experimental evidence that shall be tested under clinical conditions, in which patients treated with the combined therapy and undergoing tumor remission could be protected against any local or distant site relapses. Altogether, the evidence indicated that the chemo-vaccination strategy proposed here is a promising approach for the treatment of HPV-associated cancer likely to be further evaluated in an early-phase clinical trial.

## Supplementary Material

Supplementary figures and table.

## Abbreviations

ALT: alanine aminotransferase
ANOVA: analysis of variance
APC: antigen-presenting cell
AST: aspartate aminotransferase
BUN: blood urea nitrogen
CDDP: cis-diamminedichloroplatinum
DC: dendritic cell
EGFR: epidermal growth factor receptor
FBS: fetal bovine serum
gD: glycoprotein D
GSH: glutathione
GSSG: oxidized glutathione
HSV-1: herpes simplex virus 1
IGFR: insulin-like growth factor receptor
H&E: hematoxylin and eosin
HPV: human papillomavirus
IM: intramuscular
IV: intravenous
mAbs: monoclonal antibodies
MDSC: myeloid-derived suppressor cell
MPEC: memory precursors effector cell
PBS: phosphate buffered saline
MΦ: macrophage
pIC: poly(I:C)
SC: subcutaneous
SLEC: short-lived effector cell
SPC: second primary cancer
US FDA: United States Food and Drug Administration
