# Supplementary material for: Active immunization combined with cisplatin confers enhanced therapeutic protection and prevents relapses of HPV-induced tumors at different anatomical sites
Source: Int J Biol Sci. 2022 Jan 1;18(1):15–29. doi: 10.7150/ijbs.56644 (PMC8692155; doi:10.7150/ijbs.56644)
Supplement: Supplementary file 1 — Supplementary figures and table. [file ijbsv18p0015s1.pdf]

Active immunization combined with cisplatin confers enhanced therapeutic protection and prevents relapses of HPV-induced tumors at different anatomical sites

Bruna Felício Milazzotto Maldonado Porchia[1,2,&], Luana Raposo de Melo Moraes Aps[1,2], Ana Carolina Ramos Moreno[1], Jamile Ramos da Silva[1], Mariângela de Oliveira Silva[1], Natiely Silva Sales[1], Rubens Prince dos Santos Alves[1], Clarissa Ribeiro Reily Rocha[3], Matheus Molina Silva[3], Karine Bitencourt Rodrigues[1], Tácita Borges Barros[1], Roberta Liberato Pagni[1], Patrícia da Cruz Souza[1], Mariana de Oliveira Diniz[1,2,#], Luís Carlos de Souza Ferreira[1*]

Supplementary Material

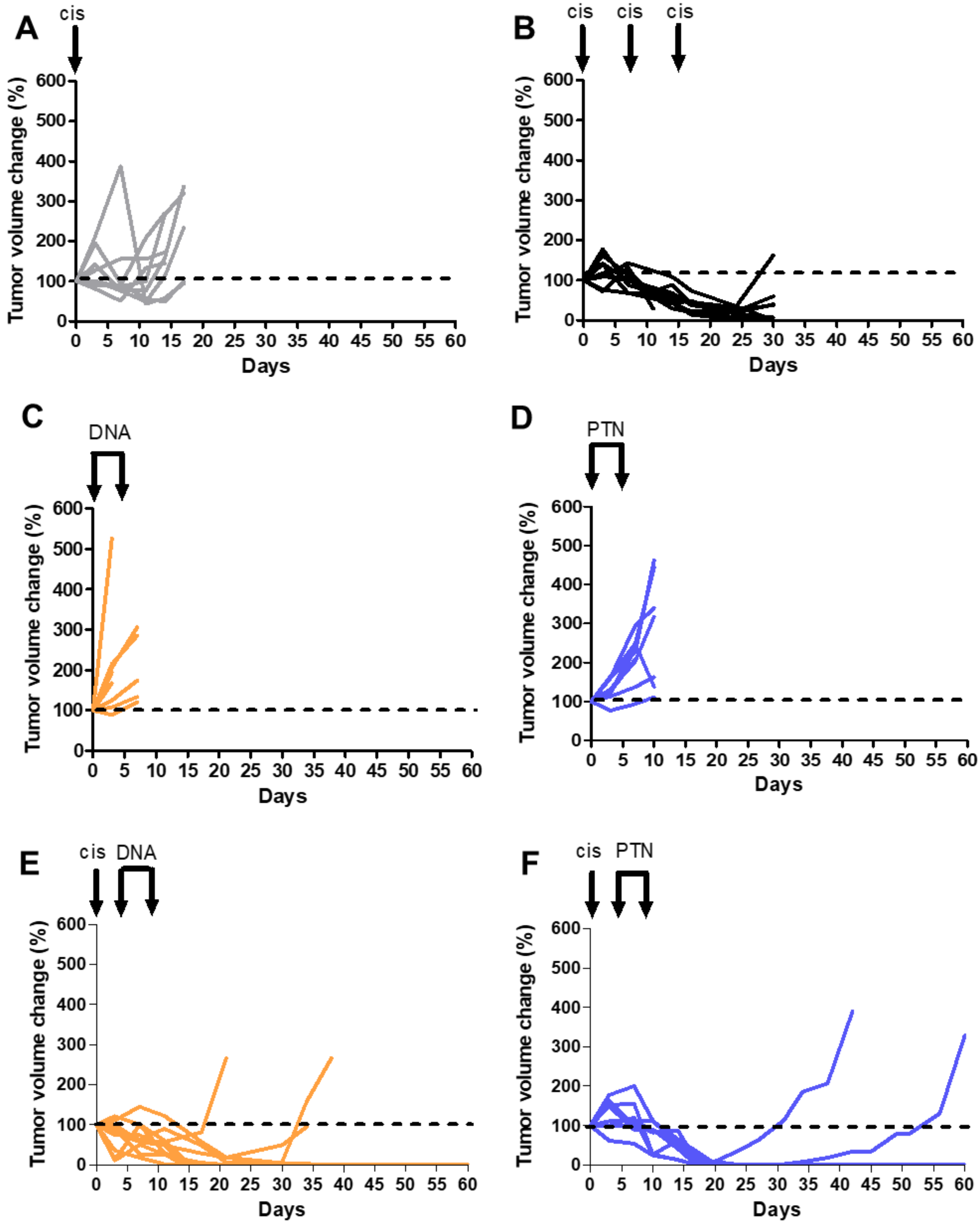
A
cis
B
cis
cis
cis
C
DNA
D
PTN
E
cis
DNA
F
cis
PTN
Tumor volume change (%)
0
100
200
300
400
500
600
Days
0 5 10 15 20 25 30 35 40 45 50 55 60

Figure S1: Combination of one dose of cisplatin with immunotherapy (pgDE7 and eletroporation or purified gDE7 and poly I:C) promotes anti-tumor effects similar to those achieved with three cisplatin doses. Individual tumor growth curves during treatment with **(A)** one or **(B)** three doses of cisplatin. Individual tumor growth curves in mice treated with pgDE7h delivered by electroporation **(C)** or purified gDE7 and poly I:C **(D)** alone. Individual tumor growth curves in mice submitted to the combined chemo/immunotherapy consisting in one dose of cisplatin followed by two doses of DNA-based **(E)** or protein-based **(F)** vaccines.

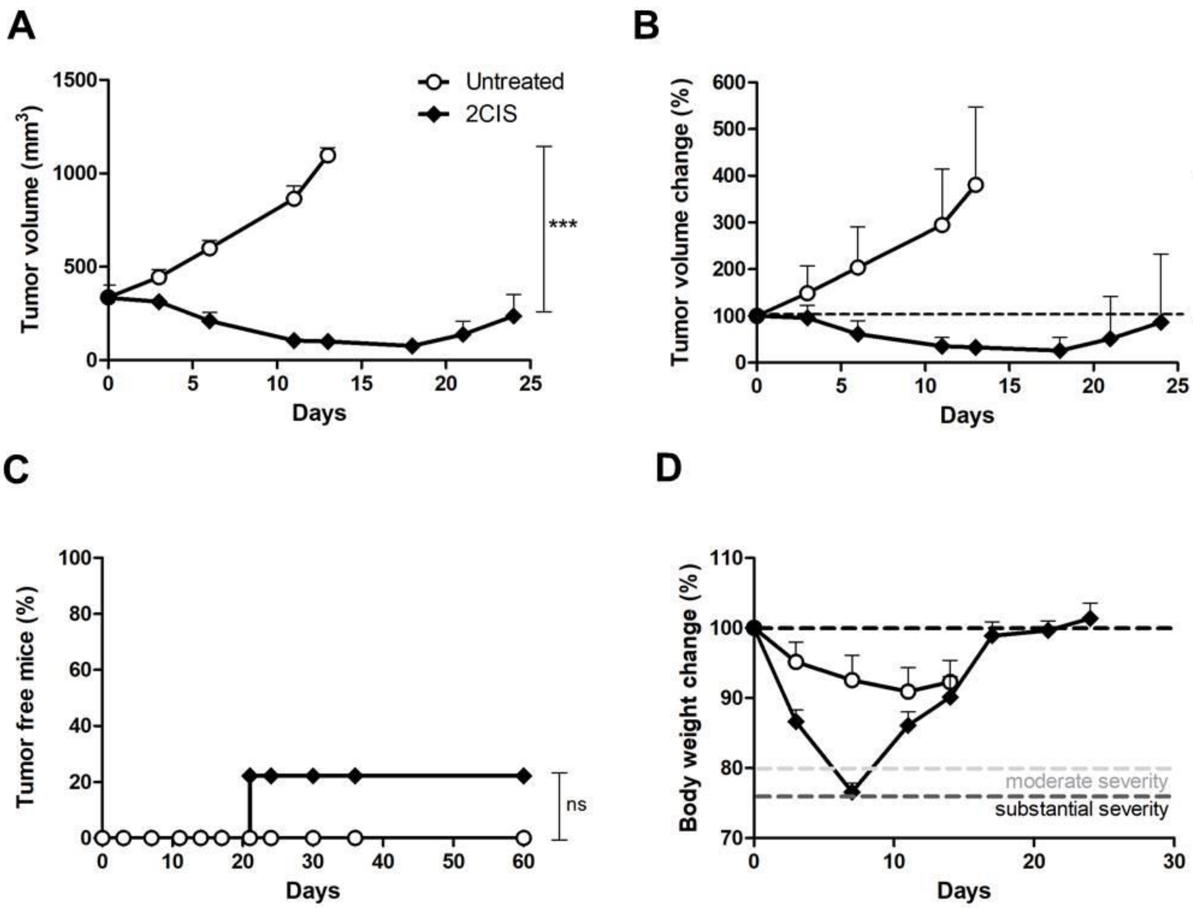

Figure S2: **Two doses of cisplatin control tumor growth but induces severe side effects. (A)** Tumor-bearing C57BL/6 mice were treated with two doses of cisplatin ("2CIS") with a 3 day interval. **(B)** Tumor volume change of the same groups. **(C)** Percentages of tumor-free mice after a follow-up period of 60 days. (**D)** Body weight change of tumor-bearing mice submitted to cisplatin treatment. Data represent the mean + SD of the combined results of two independently performed experiments. Non-treated group (n=5). "2CIS" group (n=9). Statistical significance: (**) $P < 0.01$, (***) $P < 0.001$, as determined by unpaired *t* test analysis or Log-rank (Mantel-Cox) test for tumor-free mice graph. ns: no statistical significance.

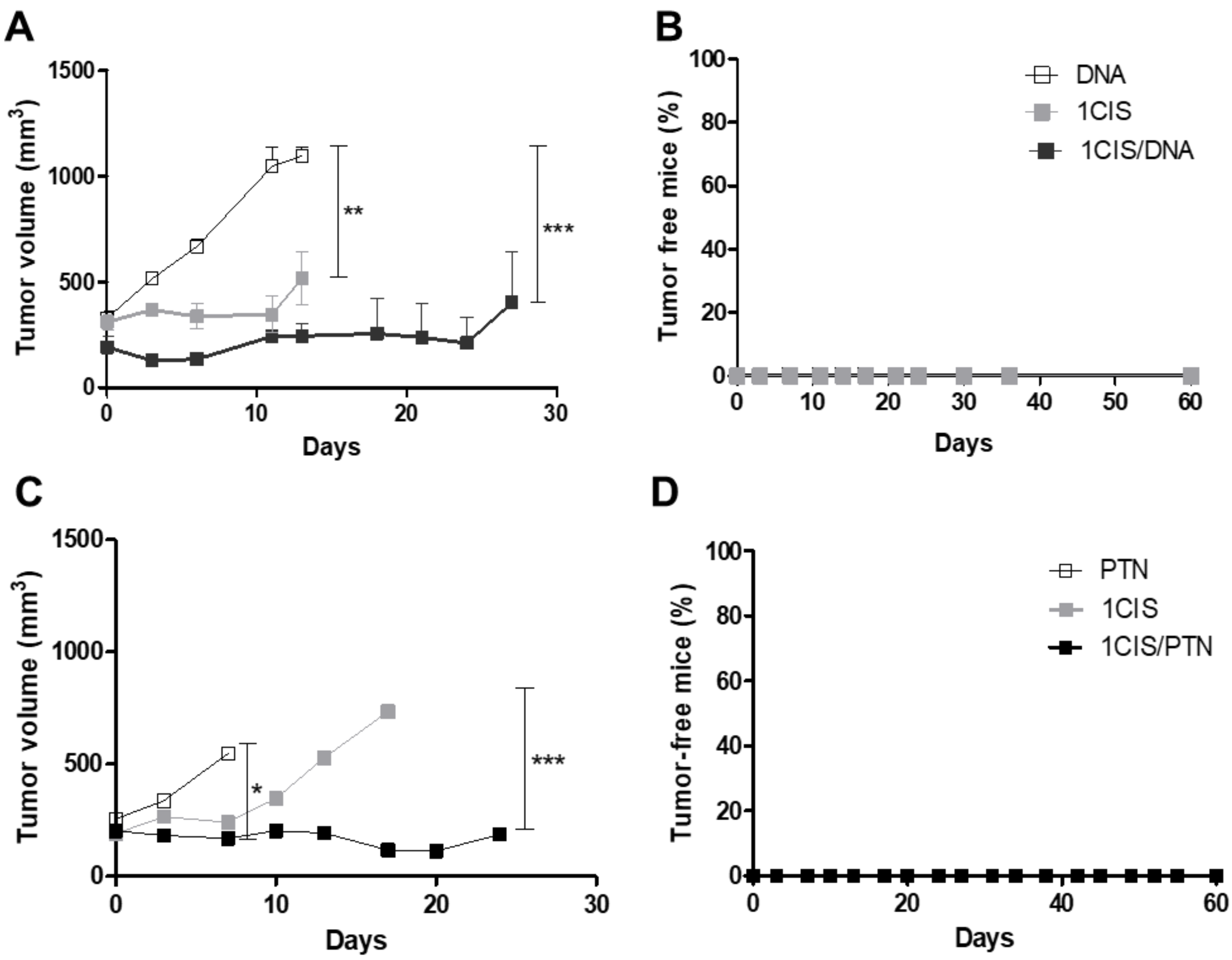

Figure S3: **Immunization with DNA without electroporation and non-adjuvanted protein associated with cisplatin controls tumor growth but does not induce synergistic effects in the SC tumor model.** C57BL/6 mice were treated with cisplatin (Day 0), IM DNA vaccine (Days 0 and 3) or SC protein vaccine without adjuvant or the combined treatment of cisplatin followed by immunotherapy (Days 0, 3 and 7) after tumors reached a diameter of approximately 10 mm. **(A)** Tumor volume (mm^3) and **(B)** Percentage of tumor-free mice treated with DNA vaccine. **(C)**, Tumor volume (mm^3) and **(D)**, Percentage of tumor-free mice treated with protein vaccine Data represent the mean of two independent experiments combined (1CIS, n=5; other groups, n=10). Statistical significance: (**) $P < 0.01$, (***) $P < 0.001$, as determined by one-way ANOVA followed by Bonferroni *post hoc* analysis.

Table S1: Histological findings in kidney and liver of mice according to treatments listed below.

Treatment	Histological findings (indicated by arrows)	
	kidney (n=3)	liver (n=3)
Naïve	Typical renal glomerulus	No histological particularities
Untreated	Typical renal glomerulus	No histological particularities
1CIS	Mild capillary congestion	No histological particularities
1CIS/DNA	Mild capillary congestion	No histological particularities
1CIS/PTN	Mild capillary congestion	No histological particularities
3CIS	Capillary thrombosis; ischemic changes	Reactive hepatocyte changes; intraparenchymal hemorrhage

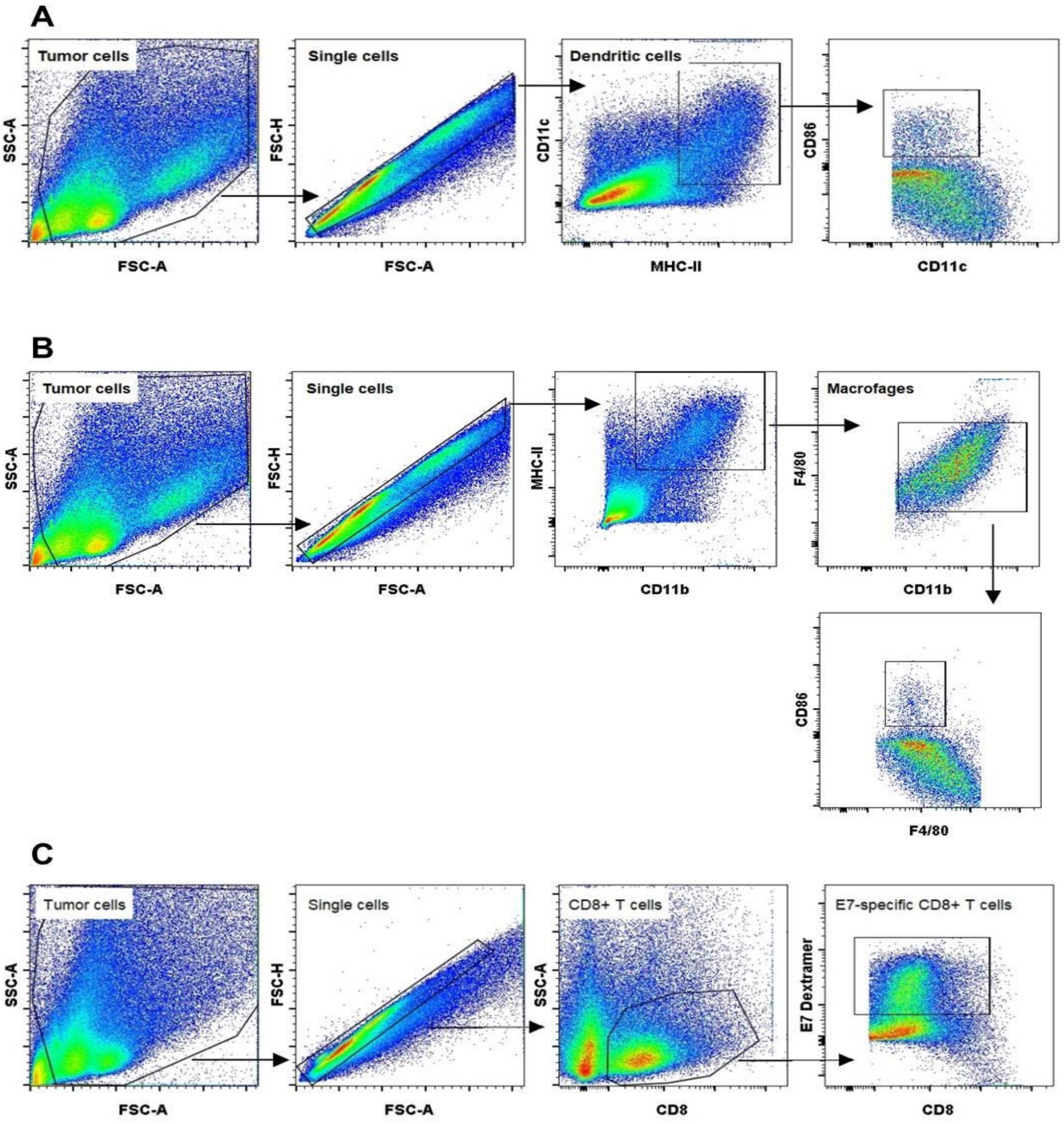

Figure S4: **Gating strategy for detection of antigen presenting cells and CD8$^+$ T cells in the tumor microenvironment.** Cells were initially gated by FSC-A and SSC-A and doublets were excluded by FSC-A and FSC-H parameters. **(A)** DCs and **(B)** macrophages were distinguished by CD11c$^+$ MCH-II$^+$ and CD11b$^+$ MCH-II$^+$ F4/80$^+$ expressions, respectively. Intratumoral DCs and macrophages were considered activated when the expression of CD86 was augmented. **(C)** The E7-specific antitumor responses were characterized by the simultaneous staining of CD8 marker and labeling with E7-specific dextramer on T cells.

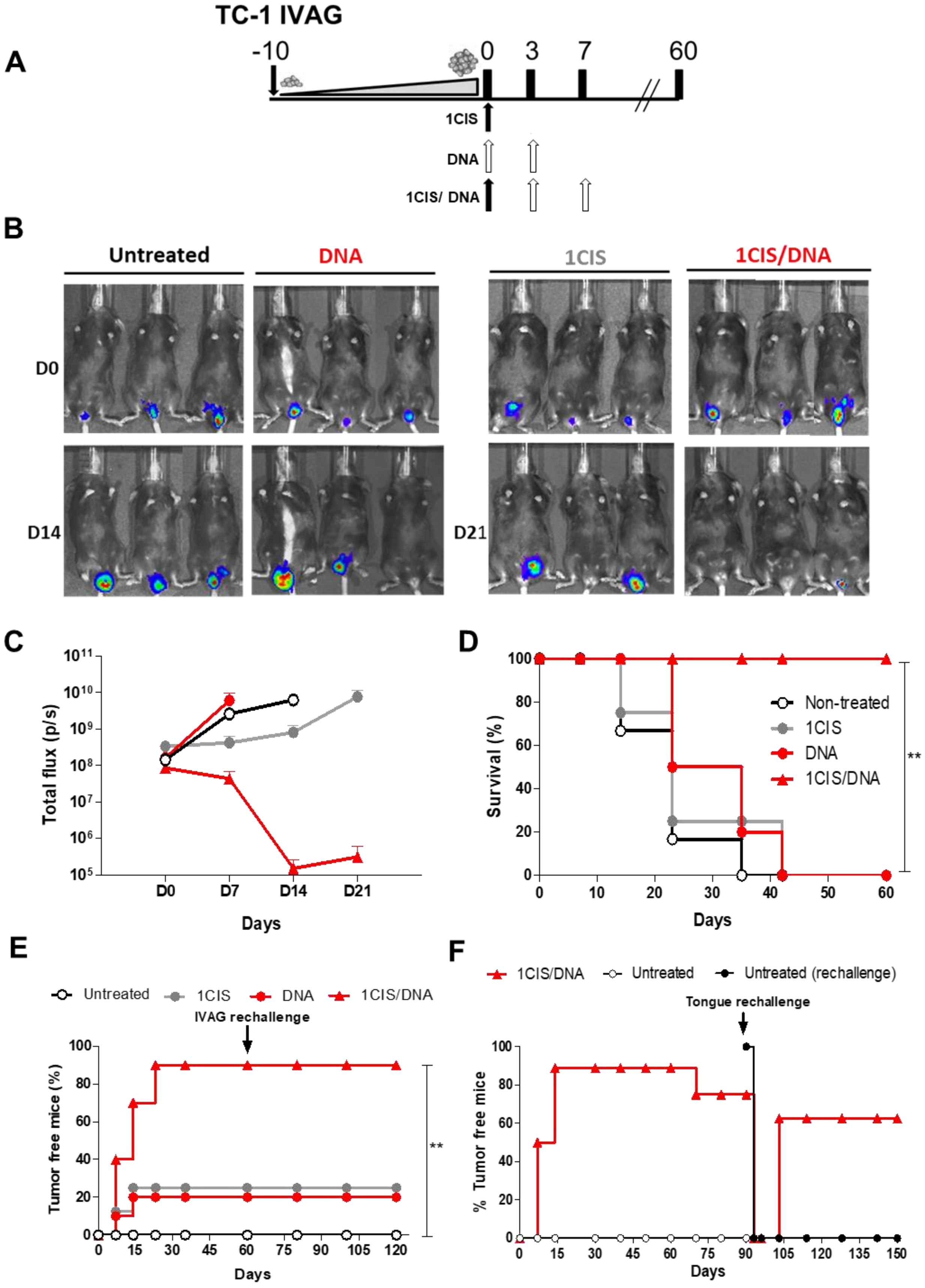
TC-1 IVAG
A
-10
0
3
7
60
1CIS
DNA
1CIS/ DNA
B
Untreated
DNA
1CIS
1CIS/DNA
D0
D14
D21
C
Total flux (p/s)
D0
D7
D14
D21
Days
D
Survival (%)
Non-treated
1CIS
DNA
1CIS/DNA
**
Days
E
Untreated
1CIS
DNA
1CIS/DNA
IVAG rechallenge
Tumor free mice (%)
**
Days
F
1CIS/DNA
Untreated
Untreated (rechallenge)
Tongue rechallenge
% Tumor free mice
Days

64

Figure S5: **Combination of cisplatin and pgDE7h-based immunotherapy confers enhanced antitumor protection to intravaginal tumors. (A)** C57BL/6 mice were inoculated with 10^5 TC-1 LUC cells intravaginally and treated with one dose of cisplatin or submitted to the pgDE7h immunization, or the combined DNA-based immunotherapy and cisplatin. **(B)** Bioluminescence images of vaginal TC-1 LUC tumors until day 14 for untreated and pgDE7h-treated (DNA) group and day 21 for mice treated with one dose of cisplatin (1CIS) and mice treated with the combined chemo(immuno)therapy (1CIS/DNA) group. **(C)** Bioluminescence emission from the tumors obtained from individual values of photons per second (p/s). **(D)** Percentage of survival and **(E)** tumor-free mice 60 days after the initial treatment followed by rechallenge with TC-1 cells (tumor relapse simulation) followed by an additional 60 day observation period. **(F)** Percentage of tumor-free mice after tongue rechallenge at day 90 followed for additional follow up period of 60 days. Data are expressed as two independently performed experiments (n=8-10). Statistical significance: (**) $P < 0.01$, as determined by Log-rank (Mantel-Cox) test.
